# A Silkworm Infection Model for In Vivo Study of Glycopeptide Antibiotics

**DOI:** 10.3390/antibiotics9060300

**Published:** 2020-06-04

**Authors:** Aurora Montali, Francesca Berini, Maurizio Francesco Brivio, Maristella Mastore, Alessio Saviane, Silvia Cappellozza, Flavia Marinelli, Gianluca Tettamanti

**Affiliations:** 1 Department of Biotechnology and Life Sciences, University of Insubria, 21100 Varese, Italy; a.montali@uninsubria.it (A.M.); f.berini@uninsubria.it (F.B.); gianluca.tettamanti@uninsubria.it (G.T.); 2 Department of Theoretical and Applied Sciences, University of Insubria, 21100 Varese, Italy; maurizio.brivio@uninsubria.it (M.F.B.); maristella.mastore@uninsubria.it (M.M.); 3 Council for Agricultural Research and Economics, Research Centre for Agriculture and Environment (CREA-AA), 35143 Padova, Italy; alessio.saviane@crea.gov.it (A.S.); silvia.cappellozza@crea.gov.it (S.C.); 4 Interuniversity Center for Studies on Bioinspired Agro-environmental Technology (BAT Center), University of Napoli Federico II, 80055 Portici, Italy

**Keywords:** insect infection model, *Bombyx mori*, glycopeptide antibiotics, vancomycin, teicoplanin, dalbavancin, insect innate immunity

## Abstract

Glycopeptide antibiotics (GPAs) are drugs of last resort for treating infections by Gram-positive bacteria. They inhibit bacterial cell wall assembly by binding to the d-Ala-d-Ala terminus of peptidoglycan precursors, leading to cell lysis. Vancomycin and teicoplanin are first generation GPAs, while dalbavancin is one of the few, recently approved, second generation GPAs. In this paper, we developed an in vivo insect model to compare, for the first time, the efficacy of these three GPAs in curing *Staphylococcus aureus* infection. Differently from previous reports, *Bombyx mori* larvae were reared at 37 °C, and the course of infection was monitored, following not only larval survival, but also bacterial load in the insect body, hemocyte activity, phenoloxidase activity, and antimicrobial peptide expression. We demonstrated that the injection of *S. aureus* into the hemolymph of *B. mori* larvae led to a marked reduction of their survival rate within 24–48 h. GPAs were not toxic to the larvae and cured *S. aureus* infection. Dalbavancin was more effective than first generation GPAs. Due to its great advantages (i.e., easy and safe handling, low rearing costs, low antibiotic amount needed for the tests, no restrictions imposed by ethical and regulatory issues), this silkworm infection model could be introduced in preclinical phases—prior to the use of mice—accelerating the discovery/development rate of novel GPAs.

## 1. Introduction

Over time, the discovery, development, and commercialization of novel antibiotics have dramatically slowed down. Thus, after the ‘golden era’ of antibiotic discovery that peaked around 1950s, the number of new antibiotics marketed each decade has declined. A recent study indicates that, between 1999 and 2014, only 25 novel antibiotics, belonging to nine chemical classes, were approved worldwide [1]. Conversely, the rapid spread of antibiotic resistance among pathogenic bacteria, considered one of the most alarming threats to global health, makes the development of novel antibacterial drugs compulsory. Notably, ESKAPE pathogens (an acronym used to indicate six multidrug-resistant, nosocomial pathogens, i.e., *Enterococcus faecium, Staphylococcus aureus, Klebsiella pneumoniae, Acinetobacter baumannii, Pseudomonas aeruginosa,* and *Enterobacter* spp.), were recently included by the World Health Organization in the list of bacteria against which new antibiotics are urgently needed [2]. 

Nowadays, many factors tend to reduce the antibiotic discovery and development success rate, from the difficulty of identifying new essential susceptible bacterial targets, to the regulatory challenges and the limited economic returns that often discourage pharmaceutical companies from investing in the field. Discovering and developing a novel antibacterial may take up to 15 years and 5000 to 10,000 candidates are screened on average, before finding a novel drug that reaches the market [3]. Although clinical trials in humans are the most challenging and expensive development phase, the need to test dozens of drug candidates in animals during preclinical selection represents the major bottleneck along the discovery process [3]. In vivo experiments using mammalian infection models (generally mice and rats) contribute to eliminating toxic compounds, selecting those better curing infections, comparing the efficacy of administration routes, and optimizing drug formulations [4]. However, the use of mammalian models is expensive and time-consuming. Specific pathogen-free facilities are needed for the growth and maintenance of experimental animals and their use is limited by ethical considerations and regulatory issues. To overcome these problems, alternative invertebrate infection models are desirable [5,6]. Although insects do not have adaptive immune systems, they have evolved innate immunity, which is highly conserved and important for resistance to microbial infections [7]. Different insects, such as *Drosophila melanogaster, Galleria mellonella,* and *Bombyx mori,* have recently been used to monitor the course and recovery from infections after antibiotic administration [8,9,10,11], and their advantages over mammalian models in terms of ethics, research costs, and speed of experimentation have become more and more evident [12].

In this paper, we investigated the silkworm *B. mori* response to infection of the Gram-positive *S. aureus*, and the subsequent administration of three clinically relevant glycopeptide antibiotics (GPAs). We selected *B. mori* as the infection model, since it is a safe and easy-to-handle insect, with reduced maintenance costs, and does not require special devices for bacterial infection and drug administration, thanks to the big size of the larvae [12]. Antibiotics can be administered to the larvae by several routes and in accurate dosage, and the following isolation of organs and hemolymph does not require particular equipment [13]. Moreover, the occurrence of standard larval instars, the synchronous development of the silkworms during the life cycle, and the reduced inter individual variability improve the reproducibility of the experiments.

**Figure 1:**
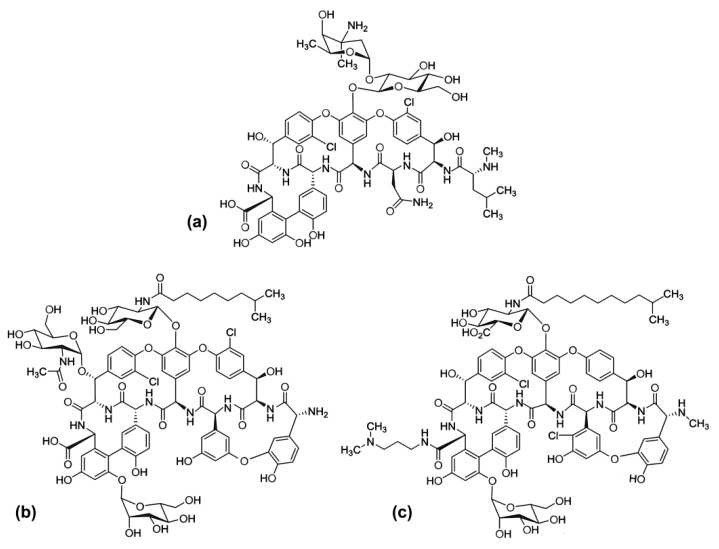
Structures of vancomycin (**a**), teicoplanin (**b**), and dalbavancin (**c**). For teicoplanin, the main component of the mixture used in clinical practice, i.e., component A2-2 bearing an 8-methylnonanoic (iso-C10:0) acid tail, is depicted.

GPAs are considered ‘drugs of last resort’ in treating severe infections by Gram-positive pathogens, such as staphylococci, enterococci, and clostridia [14,15]. Nowadays, multidrug resistant strains of *S. aureus* represent one of the major causes of mortality in hospital-acquired infections [16,17]. The GPAs tested in this study are the first-generation vancomycin and teicoplanin, natural molecules produced by filamentous actinobacteria, and the second-generation dalbavancin, obtained by chemical modification of the natural metabolite A40926 produced by *Nonomuraea gerenzanensis* [18]. Vancomycin (Figure 1a) was the first GPA approved by the US Food and Drug Administration (FDA) in 1958, whereas teicoplanin (Figure 1b) was introduced to clinical use in Europe in 1988 and in Japan in 1998. Although discovered many decades ago, they both continue to be extensively used in clinical practice. Dalbavancin (Figure 1c) was approved in 2014 and designated as Qualified Infection Disease Product by the FDA, because of its potency, extended dosing interval, and unique dose regimen [15,19]. Although only two other GPAs are today in clinical practice (telavancin and oritavancin), dozens of newly discovered semi-synthetic vancomycin and teicoplanin analogues have been described in the last few decades, and many of them are potent and promising compounds against multidrug-resistant bacterial strains, as recently reviewed in [20,21]. The primary mechanism of action of GPAs is the binding to the d-Ala-d-Ala terminus of peptidoglycan precursors of bacterial cell wall, thus blocking mature cell wall assembly and, ultimately, leading to cell lysis [22,23]. In novel derivatives, the incorporation of a new membrane depolarization and disruption mechanism improves efficacy and evades resistance [24,25,26,27].

To our knowledge, this is the first report comparing the action of three clinically relevant GPAs when administered to *B. mori* larvae infected by *S. aureus*. Different infection parameters were monitored, including the survival rate of the treated larvae and multiple immunological markers, contributing to the development of a robust and trustable infection model to be used along the discovery and development of novel GPAs.

## 2. Results

### 2.1. Rearing of B. mori Larvae at 37 °C

Under laboratory conditions, silkworms are usually reared at 25 °C [28]. With the aim to develop *B. mori* as an infection model comparable to mammalian ones, we first evaluated silkworm growth and development at 25 °C and 37 °C. We found that larval rearing at 37 °C did not produce any marked effect on the insects in the time window (from day 1 to day 4 of the fifth larval instar) considered in the experiments reported below. In fact, regardless of the incubation temperature, larvae remained mobile and actively feeding, they progressively gained weight, and their survival was not affected (100% viability in both temperature conditions). Taking into account these results, the following experiments were performed at 37 °C. 

### 2.2. Larval Survival after S. aureus Infection

**Figure 2:**
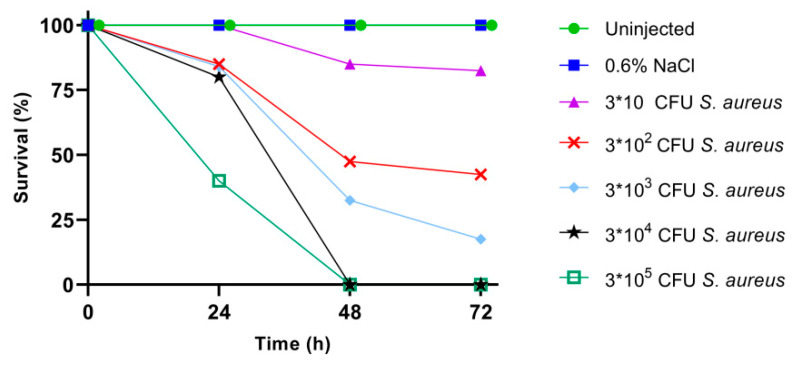
Survival rate of larvae infected with different concentrations of *S. aureus* (CFU, colony forming unit).

Different cell concentrations of *S. aureus* ATCC 6538P (from 3 × 10 to 3 × 10^5^ colony forming units (CFU) in 10 μL of injection volume) were used to infect silkworms, as described in Materials and Methods, and larval survival was monitored every 24 h for three days. In this time interval, control groups, i.e., uninjected larvae and larvae injected with only sterile physiological solution (0.6% *w*/*v* NaCl), showed normal feeding activity, and 100% of them remained viable after three days. As shown in Figure 2, 72 h after the infection, the survival of larvae infected with 3 × 10 CFU and 3 × 10^2^ CFU of *S. aureus* was 83% and 43%, respectively. Only 18% of the larvae infected with 3 × 10^3^ CFU of *S. aureus* survived at 72 h. Finally, all the larvae infected with 3 × 10^4^ CFU and 3 × 10^5^ CFU died in 48 h. These data indicate an inoculum-dependent mortality, inferring that the lethal bacterial dose which killed 50% of the infected larvae (LD_50_) corresponded to 3 × 10^2^ CFU, as estimated by Probit analysis.

### 2.3. Cure of Infected Larvae by GPA Administration

**Figure 3:**
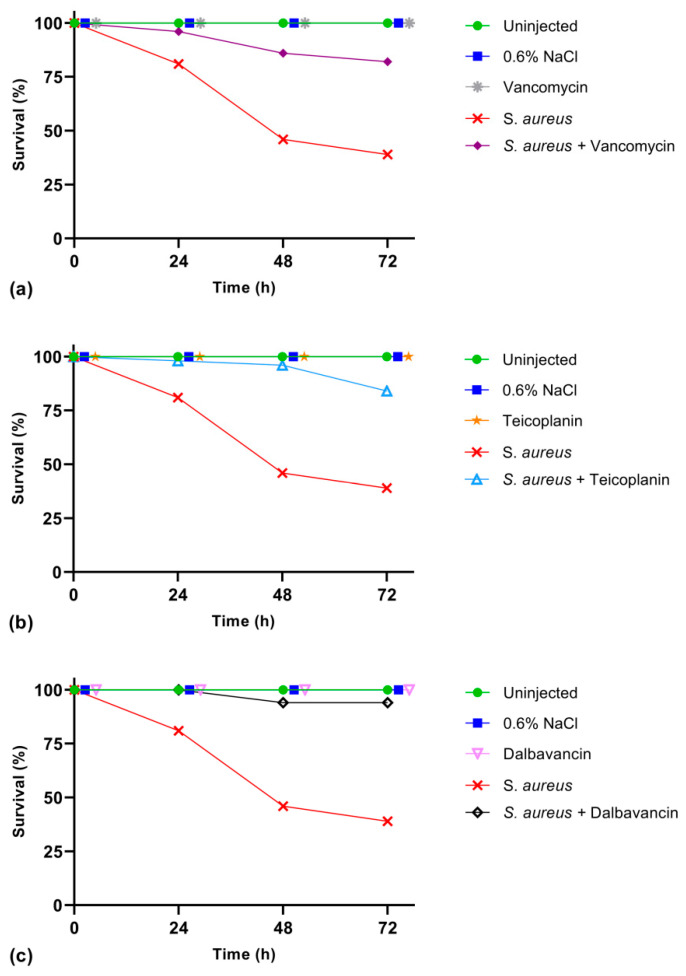
Survival rate of healthy larvae and larvae infected with *S. aureus* (3 × 10^2^ colony forming units) when treated with vancomycin (**a**), or teicoplanin (**b**), or dalbavancin (**c**). The antibiotic dose was 8.75 μg/g body weight. Results reported in (**a**–**c**) are from the same experiment run in parallel, using the same control groups (healthy larvae uninjected and injected only with saline solution) and infected larvae.

Larvae infected with *S. aureus* at LD_50_ (3 × 10^2^ CFU) were treated in parallel with the three GPAs selected for this study, i.e., vancomycin (Figure 3a), teicoplanin (Figure 3b), and dalbavancin (Figure 3c). The proper antibiotic dosage and its potential toxicity in the silkworm were initially set for vancomycin, taking into account the vancomycin antibiotic dosage (15–20 mg/kg body weight) recommended for treating bacterial infections as pneumonia, endocarditis, meningitis, etc., in humans [29]. To this purpose, three doses of vancomycin (8.75, 17.5, and 35 μg/g body weight) were injected in healthy larvae. At 72 h after the antibiotic treatment, the survival rate was 100% (data not shown), demonstrating that vancomycin was not toxic to *B. mori*. Consequently, the lowest dosage of vancomycin (8.75 μg/g body weight) was administered to healthy larvae and to infected larvae, in this last case two hours after *S. aureus* infection at LD_50_ (3 × 10^2^ CFU). Results reported in Figure 3a indicate that vancomycin was not toxic to healthy larvae, and that antibiotic administration to infected larvae significantly improved their survival rate. In fact, 81% of the infected larvae survived in the first 24 h, and this percentage decreased to 39% after 72 h, whereas the survival of larvae treated with vancomycin raised to 96% and 82% 24 h and 72 h after infection, respectively. Thus, we concluded that vancomycin can cure *S. aureus* infection in *B. mori* larvae when administered at the lowest tested dose.

The same evaluation of the potential toxicity of teicoplanin and dalbavancin in the silkworm was performed by injecting antibiotics in healthy larvae at the lowest active dose indicated for vancomycin (8.75 μg/g body weight). Similarly to vancomycin, the administration of these two GPAs did not affect larval survival, and 100% of the insects remained viable 72 h after the antibiotic treatment (Figure 3b,c). In parallel, teicoplanin and dalbavancin were injected in infected larvae at the same concentration as vancomycin (8.75 μg/g body weight), two hours after *S. aureus* infection. The administration of teicoplanin increased the survival rate of infected larvae to 98% after 24 h and to 84% after 72 h from the injection (Figure 3b), whereas dalbavancin was even more effective considering that all the larvae survived 24 h after the antibiotic administration and 94% of them survived at 72 h (Figure 3c).

These results on the curing effect of the three GPAs in vivo are in agreement with their minimum inhibitory concentration (MIC) and minimum bactericidal concentration (MBC) towards the *S. aureus* strain used in this study (Table 1). Dalbavancin (the novel semi-synthetic second-generation GPA) was more effective either in vivo or in vitro than teicoplanin and vancomycin, validating *B. mori* as a trustable infection model. These data are in fact consistent with those previously reported on these GPAs in preclinical and clinical studies in mice and humans, respectively [30,31] (see also Discussion).

### 2.4. GPA Effect on Bacterial Load in the Larvae

**Figure 4:**
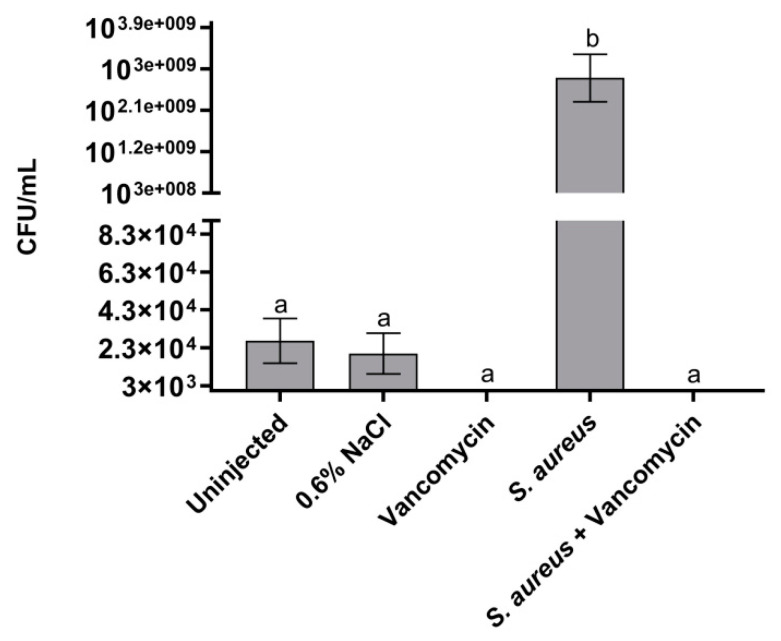
Analysis of bacterial load in control larvae (uninjected larvae and larvae injected only with physiological solution) and in healthy and infected larvae treated and untreated with vancomycin (8.75 μg/g body weight). Values represent mean ± s.e.m. Different letters indicate statistically significant differences among treatments (*p* < 0.05).

The effect of bacterial infection and GPA administration in *B. mori* was also followed by measuring the bacterial load in the silkworm body. As in previous experiments, larvae were infected with *S. aureus* at LD_50_ (3 × 10^2^ CFU) and the administration of each GPA at 8.75 μg/g body weight followed two hours after the infection. As shown in Figure 4, the bacterial load, measured as described in Materials and Methods, was comparable in the two control groups, i.e., uninjected larvae and larvae injected only with physiological solution, and this was likely due to the endogenous bacterial biota of the larvae. In the larvae infected with *S. aureus*, the bacterial load measured 24 h after the infection was very high (*ca*. 2.8 × 10^9^ CFU/mL), indicating that *S. aureus* replicated quickly in the insect. The GPA administration (vancomycin in Figure 4) to the infected larvae reduced to zero the bacterial load in the insect body, confirming the antibiotic efficacy. Interestingly, the administration of the antibiotic alone to uninfected larvae exerted a bactericidal effect on the silkworm microbiota; thus, it would be interesting to further investigate the composition of endogenous microbiota and its response to GPAs and other antibiotics in the present *B. mori* infection model [32]. 

### 2.5. Immunological Markers of Infection

As other insects, *B. mori* possesses both humoral and cellular immune responses [33]. Defensive processes involving cell-mediated phagocytosis, encapsulation, and nodulation, are usually coupled with non-self-mediated melanization by activation of prophenoloxidase system (proPO), and antimicrobial peptide production triggered by bacterial infection [10]. In this study, we followed the cellular and humoral *B. mori* responses during *S. aureus* infection and GPA administration in the experimental model set up above (infection of the larvae with *S. aureus* at LD_50_, followed 2 h later by GPA administration at 8.75 μg/g body weight). 

#### 2.5.1. Hemocyte Activity

**Figure 5:**
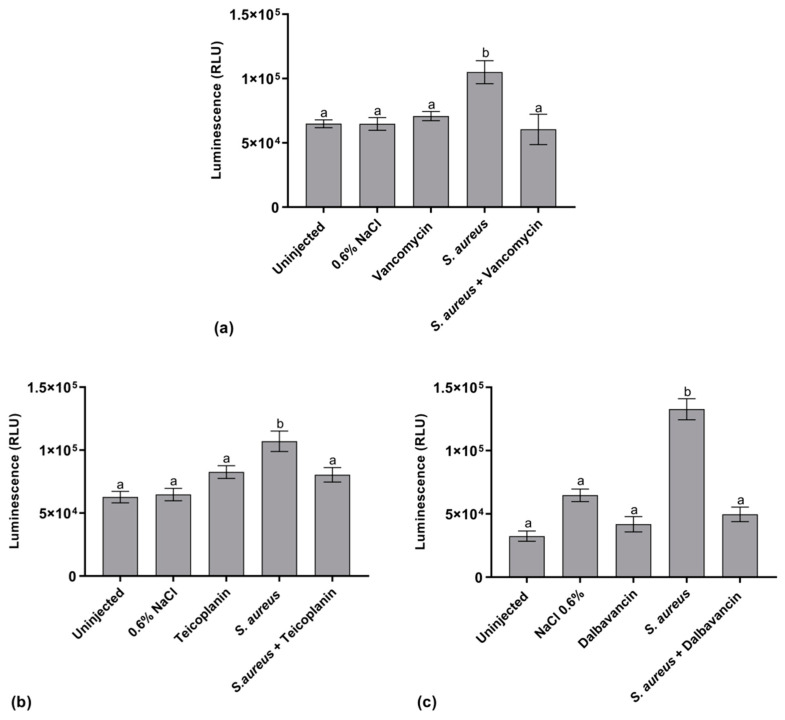
Luminescence indicating hemocyte activity in control larvae (uninjected larvae and larvae injected only with physiological solution) and in healthy and infected larvae treated and untreated with vancomycin (**a**), teicoplanin (**b**), and dalbavancin (**c**) at 8.75 μg/g body weight. Values represent mean ± s.e.m. Different letters indicate statistically significant differences among treatments (*p* < 0.05).

A luminescence assay, based on ATP content quantification, was set up for evaluating the activity of hemocytes, which are responsible for the immune cell response against pathogen invasion [34]. Our results show a significant enhancement of ATP levels in infected silkworms (Figure 5), indicating an increased metabolic activity of immune cells induced by the exposure to *S. aureus.* The luminescence value in infected larvae treated with GPAs was comparable to that observed in uninfected control groups (i.e., uninjected larvae, larvae injected with the physiological solution, and larvae injected only with the GPA), suggesting that the antibiotic, killing *S. aureus,* blocks hemocyte activity/recruitment. The response in hemocyte activity following administration of vancomycin (Figure 5a), teicoplanin (Figure 5b), or dalbavancin (Figure 5c) was statistically comparable.

#### 2.5.2. AMP Expression

**Figure 6:**
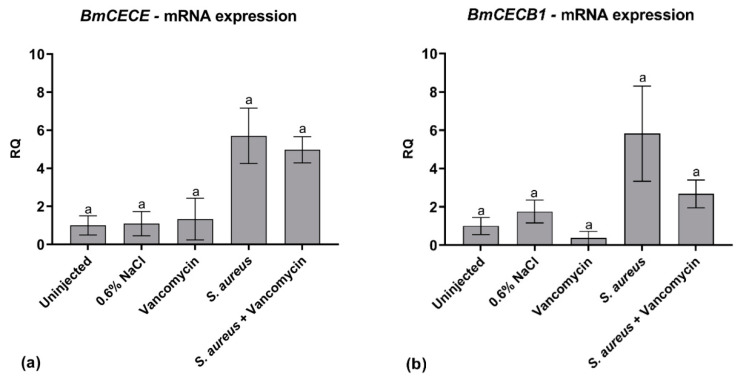
Quantitative reverse transcription PCR (qRT-PCR) analysis of *BmCECE* (**a**) and *BmCECB1* (**b**). mRNA levels in control larvae (uninjected larvae and larvae injected only with physiological solution) and in healthy and infected larvae treated and untreated with vancomycin (8.75 μg/g body weight). Values represent mean ± s.e.m. Different letters indicate statistically significant differences among treatments (*p* < 0.05).

Antimicrobial peptides (AMPs) are naturally occurring molecules produced as a first line of defense against pathogenic infections. They play an essential role in those organisms that base their defense only on innate immune response, such as insects [35]. In the silkworm, mRNA synthesis of several AMPs, as cecropins, is highly upregulated as soon as the larvae undergo bacterial infection [36]. Herein, we monitored mRNA transcription of *BmCECE* and *BmCECB1* genes coding for silkworm cecropins, which are reported to be active against Gram-positive and Gram-negative bacteria [35]. For both genes, no significant differences in mRNA levels were observed in all the control groups of larvae. Apparently, the expression level of the two AMPs increased in the infected larvae, as expected, and then it was (*BmCECB1*), or it was not (*BmCECE*), reverted to the basal level by GPA administration (vancomycin in Figure 6), but these results were not statistically significant. Further investigations are needed to understand the relatively low and variable level of AMP gene expression observed in these experiments after infection (see Discussion), and how antibiotic administration might counteract it.

#### 2.5.3. Activation of the proPO System

**Figure 7:**
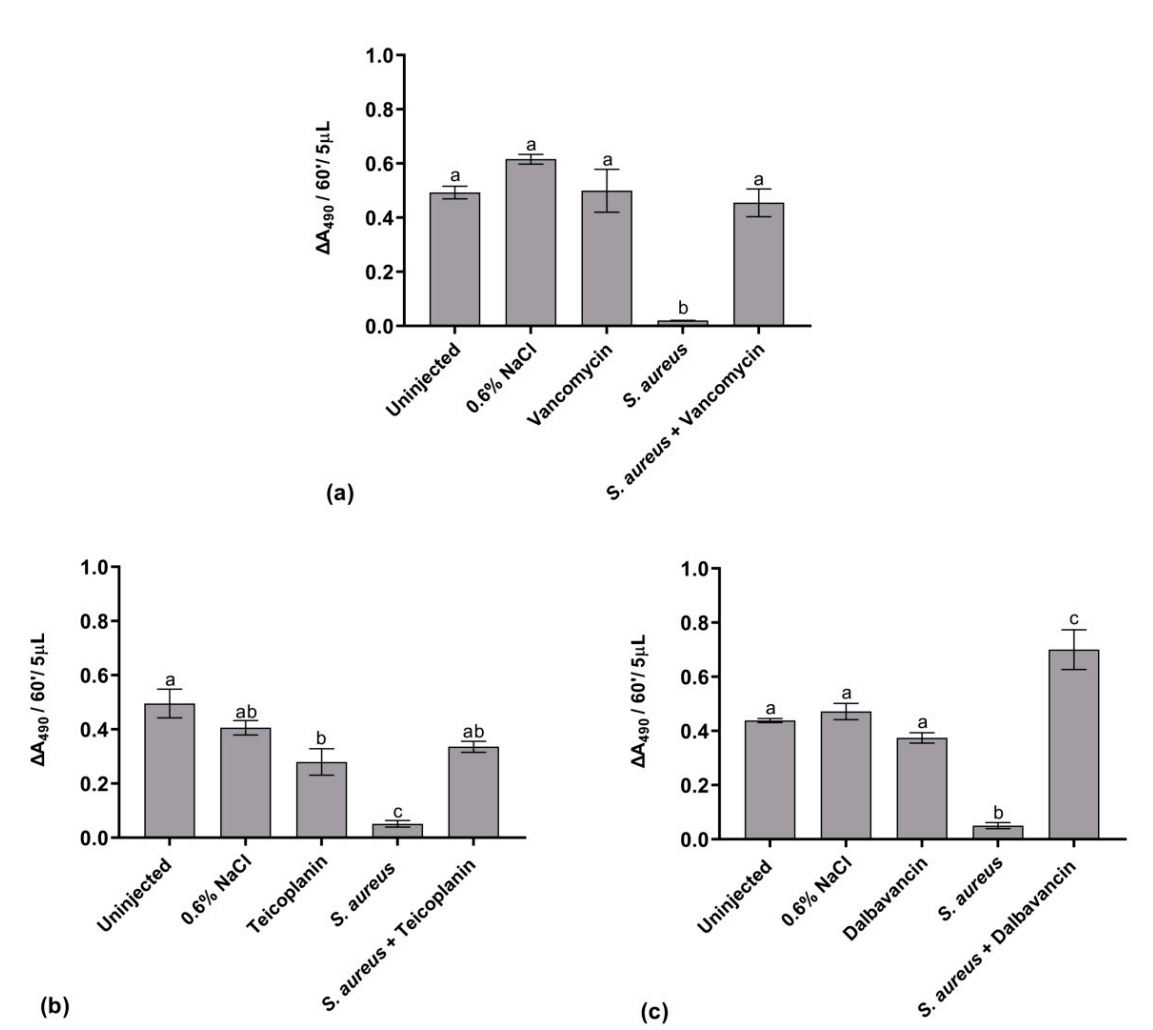
Analysis of phenoloxidase (PO) activity in control larvae (uninjected larvae and larvae injected only with physiological solution) and in healthy and infected larvae treated and untreated with vancomycin (**a**), teicoplanin (**b**), and dalbavancin (**c**) at 8.75 μg/g body weight. Values represent mean ± s.e.m. Different letters indicate statistically significant differences among treatments (*p* < 0.05).

In insects, proPO system activation and the consequent synthesis of melanin are considered important mechanisms of the immune response. Melanization is often induced by pathogens that enter the host [37]. Herein, the activation of the proPO system was monitored by measuring the phenoloxidase (PO) enzyme activity, using the enzymatic assay described in Materials and Methods. As shown in Figure 7, no significant variations in the PO relative activity were measurable in the hemolymph of the control groups. Conversely, a marked reduction of PO activity occurred following the infection of larvae with *S. aureus* at LD_50_, likely indicating a drastic impairment of immune response in the presence of high bacterial load in the hemocoel. The administration of vancomycin (Figure 7a) or teicoplanin (Figure 7b) to infected larvae restored the basal level of PO activity. Indeed, after dalbavancin administration, the level of PO activity was enhanced if compared to control conditions (Figure 7c). Thus, restoration of PO activity seems dependent on the curing effect of antibiotics. Although the physiological meaning of these observations remains to be further investigated, the PO assay might be promising for evaluating the efficacy of different GPAs.

## 3. Discussion

*B. mori* has long been used for silk production [38] and it represents an established biological model for studying insect physiology and immunity [39,40]. More recently, *B. mori* has been proposed as a model of infection alternative to mammalian ones [41,42]. Larvae infected by those pathogenic bacteria or fungi that are fatal in humans generally die, but their infection could be counteracted by antibiotic administration [12,43]. Efficacy of antibacterial agents belonging to different chemical classes, including kanamycin, tetracycline, fluconazole, etc., was previously tested in silkworm larvae [41,42,44,45]. Most of these studies monitored the larval survival, the proliferation of the infecting bacterium in the larval body, and the pharmacokinetics of antimicrobial agents [41,42,46]. Effects on the innate immunity of silkworm remained largely unexplored. In this work, we studied *B. mori* as an infection model to evaluate the efficacy of old and novel GPAs, monitoring a set of markers spanning from larval survival to cellular and humoral immunological responses. 

First, we demonstrated that *B. mori*—as in the case of *G. mellonella*, which is already used as an invertebrate infection model [8,47]—has larval stages that can survive at 37 °C, allowing the study of microbial virulence under human basal temperature. Although a negative effect of high temperatures on silkworm survival rate was previously documented [48], herein, we set up tightly controlled experimental conditions for larval growth, such as the exposure to 37 °C for a short timeframe of the fifth larval instar, and the use of a germ-free artificial diet to feed the larvae [28]. Silkworm reared on artificial diet, instead of mulberry leaves, show a reduction of the gut microbiota diversity [49]. Larvae with such microbiota are less prone to events of gut flora imbalance and secondary bacterial septicemia, which could be induced by high temperatures, and that can decrease silkworm resistance, immunity, and survival [50]. Additionally, this simplified endogenous bacterial biota might favor a better evaluation of bacterial virulence in the silkworm, thus improving the robustness of our infection model.

Our results of larval response to the infection showed a direct correlation between *S. aureus* inoculum and the survival rate. Overall, we observed a lower survival rate of infected larvae if compared to previous studies [41,42], in which silkworms were reared at 25 °C. The different temperature at which larvae where reared could explain this dissimilarity. Indeed, the rearing temperature used in this study (i.e., 37 °C) corresponds to the optimal growth temperature for *S. aureus*, determining a higher bacterial proliferation in the hemocoel and compromising larval vitality just a few hours after the infection. The administration of GPAs at dosages comparable to the ones used in humans was effective in treating infected larvae, and in significantly reducing the bacterial load in the larval body. The silkworm infection model, as set in our experimental design, turned out to be predictive of the higher and more prolonged potency of the second generation dalbavancin compared to the first-generation vancomycin and teicoplanin. Although future investigations will focus on varying GPA dosage and/or administering them in repeated injections, the in vivo and in vitro data herein reported are in agreement with those previously highlighted during preclinical studies in rats and mice, and clinical trials in humans [19,31]. When used for treating staphylococcal endocarditis in rats and septicemia in immunocompetent and neutropenic mice, a single daily dose of dalbavancin was found to be equal, or even more effective, than multiple doses of either teicoplanin or vancomycin [30]. A higher efficacy of dalbavancin was also demonstrated during phase II and phase III clinical trials in adult patients with catheter-related staphylococcal bloodstream infection, or affected by skin and soft-tissue infections [31,51].

In the second part of our work, we investigated the activation of innate cellular and humoral responses of *B. mori* to *S. aureus* infection. Our data confirmed the primary role of hemocytes against pathogen invasion [34] and indicated that the activity of these cells might be used as a marker for monitoring bacterial infection and GPA administration in silkworm. The involvement of the humoral response was assessed studying variations in PO activity and expression of AMPs [52,53]. The relative activity of PO turned out to be a good indicator for monitoring infection progress and for evaluating the curing activities of diverse GPAs. Indeed, the analyses of gene expression pattern for silkworm cecropins did not provide the expected results. In fact, although an apparent increase in the transcriptional rate was observed in infected silkworms compared to controls, the difference registered among the experimental groups could not be considered statistically significant. Romoli et al. (2017) demonstrated that AMP production after bacterial infection depends on the silkworm strain and pathogen in use [36]. AMP production is lower in Chinese and Japanese silkworm strains. The reduced expression level of the two AMP genes that we observed 24 h after infection could be due to the use of polyhybrid silkworms, derived from a four-way crossbreed between a Chinese and a Japanese strain. However, we cannot exclude that the unexpected variations of AMP mRNA expression levels are somehow due to the high rearing temperature, as previously shown for other genes [54]. 

Although further investigations on the role and interaction of the different components concurring to the innate immunity of *B. mori* are needed, our work adds novel evidence on the current knowledge. We could identify useful tools for monitoring bacterial infection and evaluating GPA therapeutic potential in this simple, inexpensive, and easy-to-handle insect model. Measurements of larval survival rate, hemocyte activity, and PO enzyme activity in infected larvae treated and untreated with different antibiotics were easy to perform and relatively cheap; moreover, they required low amount of GPAs to be tested, and allowed us to assess the potency of three clinically important GPAs. Thus, this study contributes to validating *B. mori* as an alternative animal infection model for screening old and novel GPAs, potentially reducing the number of mammals to be used in the preclinical phases of drug discovery and development. This outcome may be relevant, considering that novel GPAs are urgently needed to overcome emerging antimicrobial resistance and prolong the clinical longevity of this important antibiotic class [14,15,17,20,21].

## 4. Materials and Methods

### 4.1. Experimental Model

Larvae of *B. mori* [polyhybrid (126 × 57) (70 × 90)], provided by CREA-AA, Sericulture lab (Padova, Italy), were reared in groups of ten in glass Petri dishes (180 mm × 30 mm) at 25 ± 0.5 °C under a 12:12 h light:dark period and 70% relative humidity. Larvae were fed on artificial diet [28] until the end of the IV larval instar. After animals had ecdysed to the V larval instar, silkworms were synchronized [55] and fed with a daily amount of antibiotic-free and germ-free artificial diet, as previously reported in [56]. All the experiments were performed with larvae at fifth instar.

### 4.2. B. mori Rearing at 37 °C

After the last larval molt, silkworms were incubated at 37 ± 0.5 °C and grown as described in Section 4.1. Insect growth, mortality, and larval behavior were assessed daily during the fifth larval instar and data were compared to those obtained from larvae grown at 25 ± 0.5 °C. Thirty larvae were used for each temperature condition.

### 4.3. Bacterial Strains and Culture Conditions

For strain reactivation, *S. aureus* subsp. Rosenbach ATCC 6538P was grown overnight at 37 °C under shaking at 200 rpm (revolutions per minute) in 10 mL of MHB2 (Müller Hinton Broth 2, VWR International S.r.l., USA). 1 mL of culture was centrifuged for 10 min at 1900× *g* and 4 °C, supernatant was discharged, and cell pellet resuspended in sterile physiological solution (0.6 % *w*/*v* NaCl) to reach a concentration of 3 × 10^8^ CFU/mL. The volume of physiological solution to be added was calculated by measuring the optical density of the culture at 600 nm (OD_600nm_) and taking into consideration that one unit of OD_600nm_ corresponds to *ca.* 2.4 × 10^8^ CFU of *S. aureus* per mL. Serial dilutions with sterile physiological solution were then made to obtain the bacterial suspensions with the desired cell concentration.

### 4.4. Minimum Inhibitory Concentrations (MICs) and Minimum Bactericidal Concentrations (MBCs)

MICs of vancomycin (Sigma-Aldrich, St. Louis, MO, USA), teicoplanin (Sigma-Aldrich, USA), and dalbavancin (kindly gifted by Sanofi, Italy) toward *S. aureus* were determined by the broth dilution method, according to the guidelines of the Clinical and Laboratory Standards Institute [57]. The antibiotics were prepared by dissolving the corresponding powder in deionized water, filtered with a cut-off of 0.22 µm, and finally brought to appropriate dilution with MHB2. *Ca*. 5.0 × 10^5^ bacterial cells in exponential growth were inoculated in MHB2, together with increasing concentrations of the antibiotics. MICs were defined as the minimal antibiotic concentration at which no turbidity could be detected after incubation for 20 h at 37 °C and 200 rpm. For calculating MBCs, 0.1 mL of bacterial cultures used for the MIC test were plated on MHA (Müller Hinton Agar, VWR International S.r.l., USA), then incubated at 37 °C for 24 h. MBCs were the lowest antibiotic concentrations, at which no growth could be observed.

### 4.5. Injection of Larvae and Collection of Hemolymph

For all the experiments described below, larvae at the second day of the fifth instar were injected in the second right proleg by using autoclaved Hamilton 1702 LT 25 μL syringes (Hamilton, USA). Injections were performed under sterile hood. Hemolymph was collected from the larva by cutting the second left proleg.

### 4.6. Determination of Lethal Dose 50 (LD_50_) for S. aureus

To determine the LD_50_, silkworms were injected with 10 μL containing different concentrations of *S. aureus* cell suspension (3 × 10^3^, 3 × 10^4^, 3 × 10^5^, 3 × 10^6^, and 3 × 10^7^ CFU/mL), and their mortality was monitored every 24 h for three days. Uninjected larvae and larvae injected with 10 μL of physiological solution (0.6% *w*/*v* NaCl) were used as controls. Forty larvae for each experimental condition were used. Larvae were considered dead when there was no reaction after stimulation with a plastic tip. LD_50_ was defined as the concentration of bacteria at which 50% of animals died within 72 h after the infection. LD_50_ and fiducial limits were calculated by Probit analysis [58].

### 4.7. GPA Administration

To evaluate the toxicity of vancomycin on the survival of healthy larvae, 10 μL of antibiotic at 8.75, or 17.5, or 35 μg/g body weight diluted in sterile physiological solution were injected and larval mortality was monitored for 72 h. Uninjected larvae and larvae injected with 10 μL of physiological solution (0.6% *w*/*v* NaCl) were used as controls. Thirty larvae were used for each experimental group. To evaluate the effects of antibiotics on the survival of infected silkworms, larvae were injected with 10 μL of *S. aureus* at LD_50_, followed by injection 2 h later of 10 μL of vancomycin, or teicoplanin, or dalbavancin at 8.75 μg/g body weight. In preliminary trials, a second injection of 10 μL of physiological solution in infected larvae was tested in parallel to antibiotic injection, and it did not alter their physiological state. Consequently, untreated (uninjected) larvae, larvae injected once with physiological solution (10 μL of 0.6% *w*/*v* NaCl), and healthy larvae injected with 10 μL of antibiotic were used as controls groups. Larval mortality was monitored for 72 h. Fifty larvae were used for each experimental condition.

To evaluate the bacterial load and the immunological markers, experimental groups were as follows: untreated larvae, larvae injected with 10 μL of 0.6% *w*/*v* NaCl, larvae injected with 10 μL of antibiotic (i.e., vancomycin, teicoplanin, or dalbavancin) at 8.75 μg/g body weight, larvae injected with 10 μL of 3 × 10^5^ CFU/mL of *S. aureus,* larvae injected with 10 μL of 3 × 10^5^ CFU/mL of *S. aureus* and, two hours later, with 10 μL of antibiotic at 8.75 μg/g body weight). After injections, silkworms were reared at 37 °C. 24 h after the first injection, surviving larvae were analyzed, as described below (Section 4.7.1, Section 4.7.2, Section 4.7.3, Section 4.7.4).

#### 4.7.1. Hemocyte Activity

Hemolymph was collected from the larvae and diluted 1:50 with Saline Solution for Lepidoptera (sucrose 210 mM, KCl 45 mM, Tris-HCl 10 mM, pH 7.0). The viability of hemocytes was evaluated by using the CellTiter-Glo Luminescent Cell Viability Assay (Promega, Madison, WI, USA). Briefly, 100 μL of diluted hemolymph and 100 μL of CellTiter-Glo reagent were mixed into a 96-well plate, and then incubated for 5 min at room temperature on an orbital shaker. Luminescence was measured using an Infinite F200 96-well plate-reader (Tecan, Männedorf, Switzerland). Ten surviving larvae for each experimental group were analyzed.

#### 4.7.2. AMP Expression

The fat body was isolated from larvae, cleaned from tracheae, and immediately frozen in liquid nitrogen. RNA was extracted from 20–30 mg of tissue with Trizol Reagent (Life Technologies, Carlsbad, CA, USA). Genomic DNA contamination was removed using TURBO DNA-free Kit (Life Technologies, USA) and the RNA quality was verified through gel electrophoresis. Primers used for quantitative reverse transcription PCR (qRT-PCR) are indicated in Table 2. *BmRP49* was used as housekeeping gene, to calculate the relative expression of the cecropin genes [59]. iTaq Universal SYBR Green Supermix (Bio-Rad, Hercules, CA, USA) and a CFX Connect Real-Time PCR Detection System (Bio-Rad, USA) were used to perform PCR. Relative expression of the genes was calculated with the 2^−ΔΔCt^ method. The efficiency of the amplification reaction of each gene was adjusted to be in the range of 90%–105%. Fifteen surviving larvae for each experimental group were analyzed.

#### 4.7.3. Activation of Prophenoloxidase System

The activation of the prophenoloxidase system was evaluated by monitoring the activity of the enzyme phenoloxidase. Hemolymph was collected from larvae and 5 μL were added to a 1 mL solution containing 8 mM L-Dopa (L-3-4 dihydroxyphenylalanine) (Sigma-Aldrich, USA) in Tris-HCl 10 mM. The relative activity of phenoloxidase (formation of dopachrome) was evaluated as an increase in optical density over time, at 490 nm for 60 min. The activity was registered using a V-560 double-beam spectrophotometer (Jasco, USA). Nine surviving larvae for each experimental group were analyzed.

#### 4.7.4. Bacterial Load

Larvae were surface sterilized with 70% *v*/*v* ethanol. They were then placed in disposable 15 mL centrifuge tubes and homogenized using a Potter-Elvehjem PTFE pestle according to [60]. 100 μL of serial dilutions of the homogenate (from 10^−1^ to 10^−5^, in sterile physiological solution) were plated onto MHA plates and incubated at 37 °C. The number of colonies (as CFU/mL) was calculated after 24 h. All the procedures were performed under sterile hood. Five surviving larvae for each experimental group were analyzed.

### 4.8. Statistical Analysis

Statistical analysis was performed using ANOVA, followed by Tukey’s Honestly Significant Difference (HSD) test (significance *p* < 0.05). 

## 5. Conclusions

Our results show the usefulness of in vivo-mimic infection of *B. mori* larvae by *S. aureus* to assess the therapeutic potential of old and novel GPAs. Immunological markers tested herein demonstrated to be promising for evaluating the efficacy of different GPAs. In particular, the novel second generation dalbavancin was confirmed to be more effective than the first-generation drugs vancomycin and teicoplanin in curing *S. aureus* infection. Our hope is that this insect infection model might accelerate the discovery and development of novel GPAs, which are urgently needed to prolong the clinical longevity of such important class of life-saving drugs. This model could be introduced in preclinical phases prior to the use of mice and might help in overcoming bottleneck steps between the in vitro and in vivo experimental phases.

## Figures and Tables

**Table 1:** Minimum inhibitory concentration (MIC) and minimum bactericidal concentration (MBC) of the three glycopeptide antibiotics used in this study towards *S. aureus* ATCC 6538P. Values represent the average of data from at least three independent experiments.

Antibiotic	MIC (μg/mL)	MBC (μg/mL)
Vancomycin	1	>128
Teicoplanin	1	128
Dalbavancin	0.5	16

**Table 2:** Primer sequences used in this study.

Gene	Accession Number	Primer Sequences
*BmRP49*	NM_001098282.1	F: AGGCATCAATCGGATCGCTATG R: TTGTGAACTAGGACCTTACGGAATC
*BmCECE*	DQ233467.1	F: GTGTGTGCGAGCGTTATGGC R: CCCATGAGCGATGGTCGCC
*BmCECB1*	BGIBMGA000024-RA	F: TTCGCTCTGGTGCTGGCTTTG R: GGCCCGCTTTGACGATGCC
